# Supplementary material for: Characterization of antibiotic resistance genes in the species of the rumen microbiota
Source: Nat Commun. 2019 Nov 20;10:5252. doi: 10.1038/s41467-019-13118-0 (PMC6868206; doi:10.1038/s41467-019-13118-0)
Supplement: Supplementary file 1 — Supplementary Information [file 41467_2019_13118_MOESM1_ESM.pdf]

Supplementary Information

Antibiotic resistance genes in the species of the rumen microbiota

Sabino *et al*.

List of Contents

Supplementary Figures 1-7
Supplementary Tables 1-2

Supplementary Figures

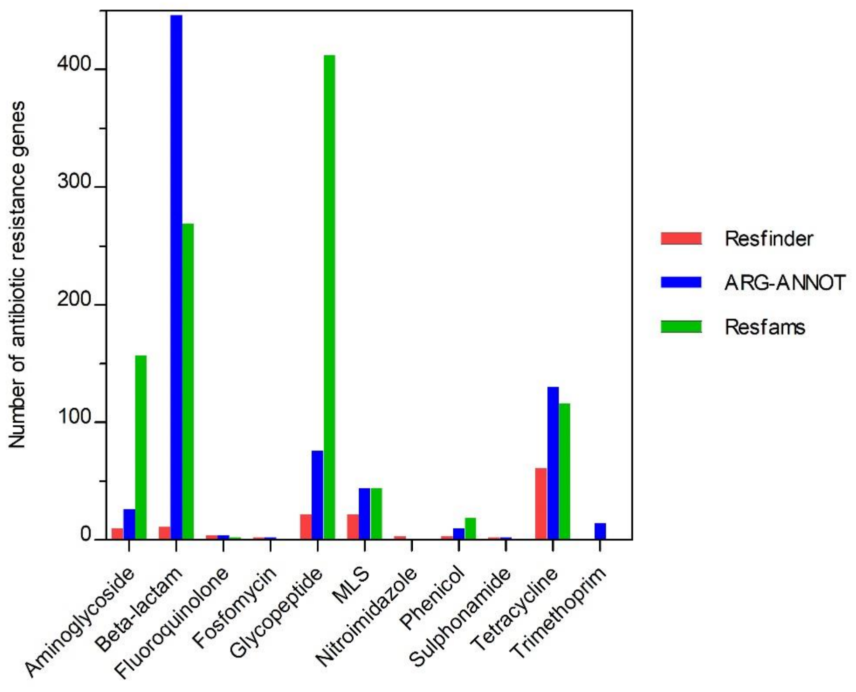

Supplementary Figure 1. Number of ARGs distributed by antibiotic class detected by ResFinder, ARG-ANNOT and Resfams in the ruminal genomes analyzed in this study. Source data are provided as a Source Data file.

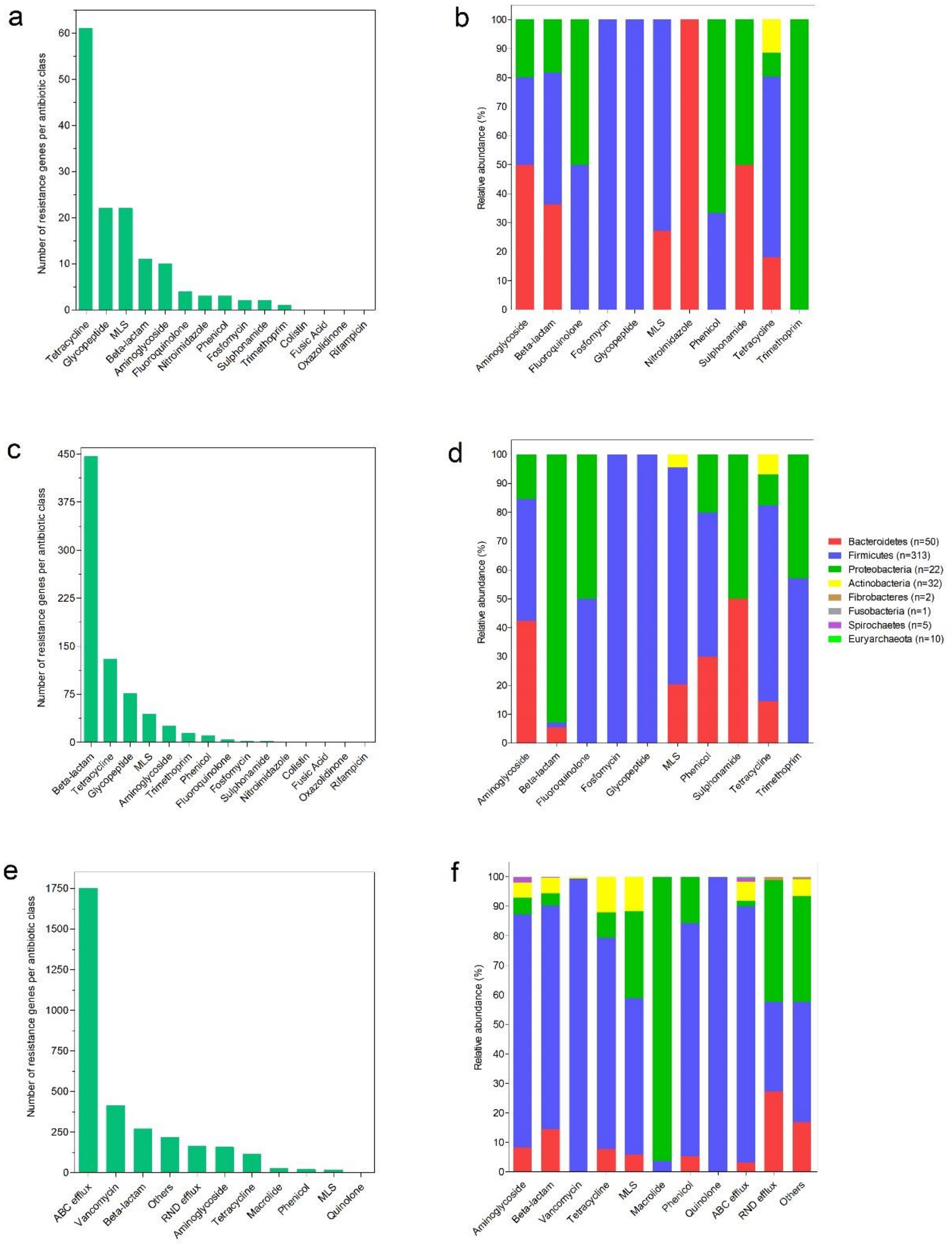

Supplementary Figure 2. Number of ARGs per antibiotic class and distribution of ARGs in the phyla analyzed in this study using ResFinder (a) and (b), ARG-ANNOT (c) and (d) and Resfams databases (e) and (f), respectively. Source data are provided as a Source Data file.

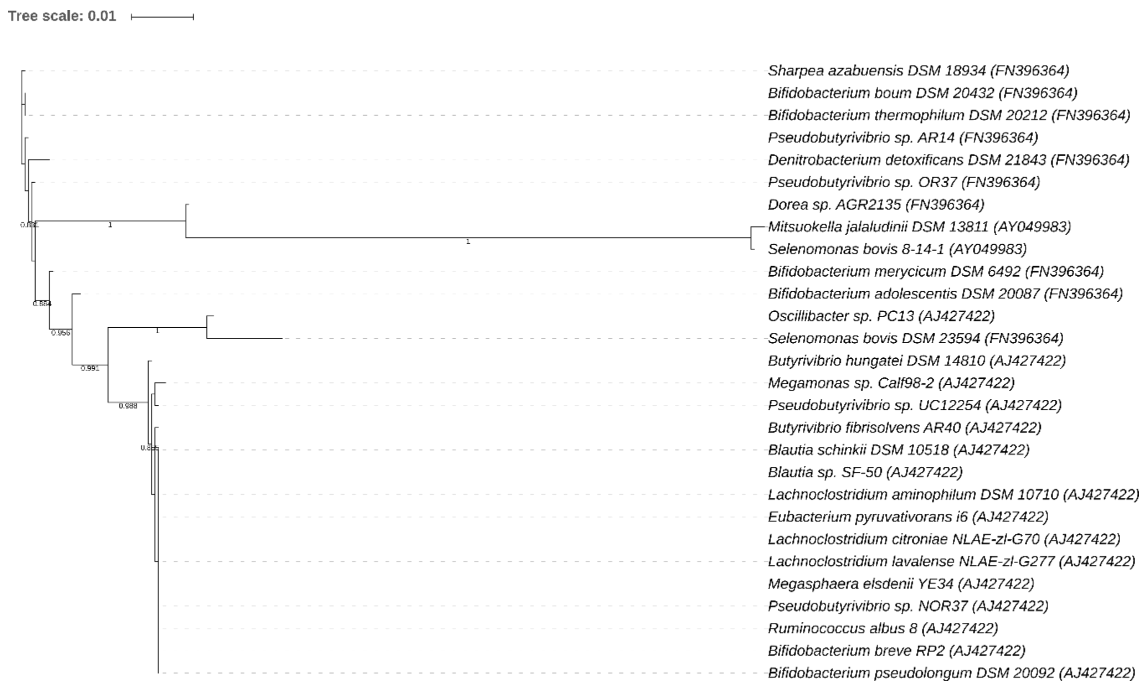

Supplementary Figure 3. Phylogenetic tree of *tet*(W) sequences identified in the genomes of ruminal bacteria. The tree was generated using FastTree (Maximum Likelihood method, 1000 replications). Gene sequences were extracted from the ruminal genomes using SAMtools. ResFinder database was used to identify the location of each *tet*(W) gene in the microbial genomes. Only bootstrap values greater than 0.7 are shown.

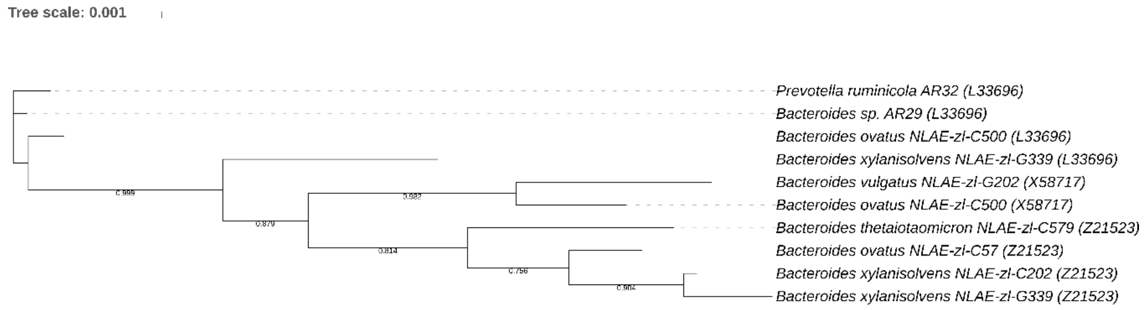

Supplementary Figure 4. Phylogenetic tree of *tet*(Q) sequences identified in the genomes of ruminal bacteria. The tree was generated using FastTree (Maximum Likelihood method, 1000 replications). Gene sequences were extracted from the ruminal genomes using SAMtools. ResFinder database was used to identify the location of each *tet*(Q) gene in the microbial genomes. Only bootstrap values greater than 0.7 are shown.

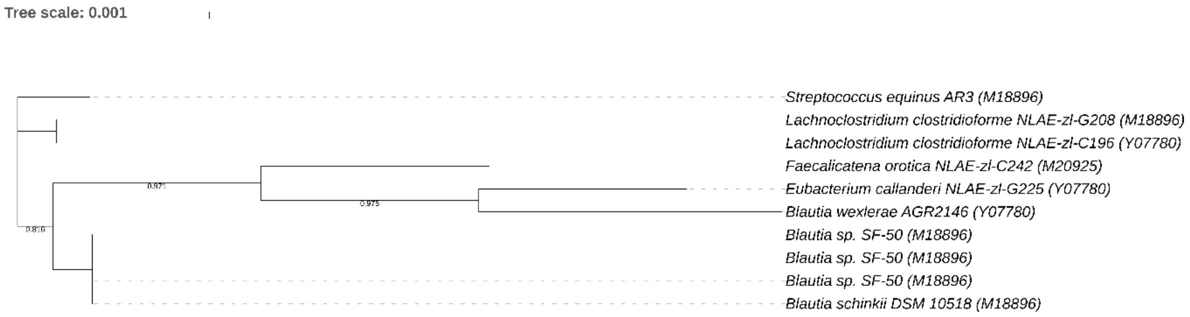

Supplementary Figure 5. Phylogenetic tree of *tet*(O) sequences identified in the genomes of ruminal bacteria. The tree was generated using FastTree (Maximum Likelihood method, 1000 replications). Gene sequences were extracted from the ruminal genomes using SAMtools. ResFinder database was used to identify the location of each *tet*(O) gene in the microbial genomes. Only bootstrap values greater than 0.7 are shown.

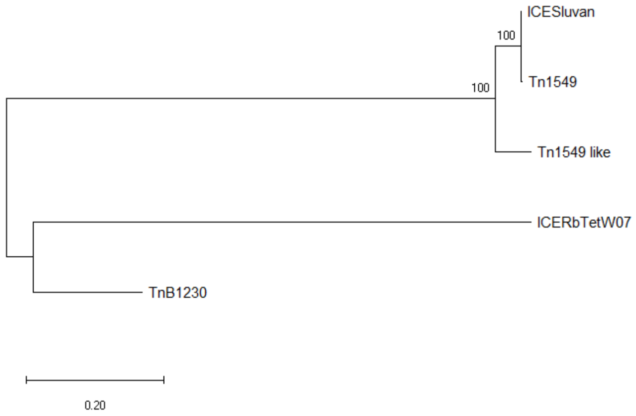

Supplementary Figure 6. Phylogenetic comparation of the ICE DNA sequence from ICE_*RbTetW_07* (identified in this work in *Blautia schinkii* 10518), TnB1230 from *Butyrivibrio fibrisolvens*, Tn1549 from *Enterococcus faecalis*, Tn1549-like from *Clostridioides difficile* and ICESluvan from *Enterococcus faecium*. The sequences were aligned using Muscle and the phylogenetic tree was reconstructed using the Maximum Likelihood method with 100 replication, both in MEGAX software. The branch lengths represents the number of substitutions per site.

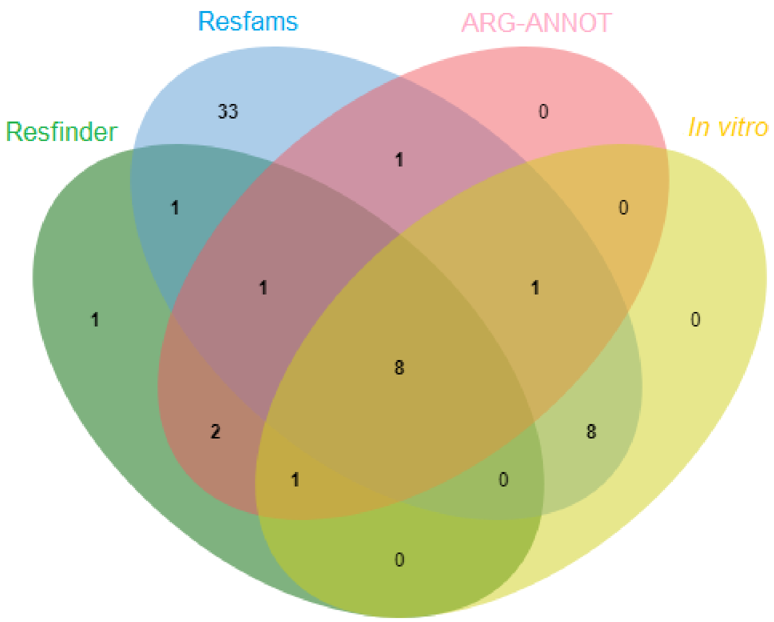

Supplementary Figure 7. Venn diagram showing the number of ARGs (ARG-ANNOT, ResFinder and Resfams) or resistance phenotypes (detected *in vitro*) identified in the 26 ruminal genomes or corresponding cultures. The Venn diagram was constructed using the tools available in the "Bioinformatics & Evolutionary Genomics" website (http://bioinformatics.psb.ugent.be/webtools/Venn/). Source data are provided as a Source Data file.

Supplementary Tables

Supplementary Table 1. Susceptibility of ruminal bacteria to different classes of antibiotics. Antimicrobial resistance was evaluated by determining the MIC (Minimum Inhibitory Concentration) using the Epsilometer test (E-test) method. Results were interpreted according to the established EUCAST breakpoints.

Bacteria	BLA		AMG		**Tetracycline**[1]	**Vancomycin**	**Chloramphenicol**	MAC[1]		Clindamycin
	Ampicilin	Amoxicillin	Gentamicin[3]	Streptomycin*				Erythromycin	Azithromycin	
Acetitomaculum ruminis DSM 5522						S				S
Bifidobacterium adolescentis DSM 20087	S	S			R					
Bifidobacterium boum DSM 20432	S	S			R					
Bifidobacterium merycicum DSM 6492			S	4	S					
Bifidobacterium pseudolongum DSM 20092			R	64	R					
Bifidobacterium thermophilum DSM 20212					R					
Blautia schinkii DSM 10518	S	S	S	12	R	R				
Butyrivibrio fibrisolvens DSM 3071			S	4		S				
Butyrivibrio proteoclasticum B316			S	4		S				
Butyrivibrio sp. M55	S	S				S				
Lachnoclostridium aerotolerans DSM 5434	S	S	S	8	S	S	S			
Clostridium intestinale DSM 6191	S	S			S	R				
Clostridium lundense DSM 17049	S	S				R	S	R	S	
Lachnoclostridium polysaccharolyticum DSM 1801						R				
Lachnoclostridium aminophilum DSM 10710	S	S			R	S				
Lachnospira multiparus D15d	S	S								
Lactobacillus ruminis DSM 20403						R				
Megasphaera elsdenii T81	S	S								
Mitsuokella jalalundinii DSM 13811	R	R			R					
Olsenella umbonata DSM 22619	S	S								
Prauserella rugosa DSM 43194	S	S						R	R	
Proteiniclasticum ruminis DSM 24773	S	S	I	8		S[2]		S	S	S
Ruminococcus flavefaciens FD-1						S				
Selenomonas ruminantium DSM 2872	R	R								
Sharpea azabuenensis DSM 18934	S	S			R	S				
Sharpea azabuenensis DSM 20406	S	S				S				

*Numbers represent the MIC values (µg/ml) for streptomycin since no interpretative standards are available for this antibiotic in EUCAST; [1]BV for *Streptococcus* sp.; [2]BV for Gram positive bacteria; [3]BV for Enterobacteriaceae. R = resistant; S = susceptible; I = intermediate; BLA = beta-lactam; AMG = aminoglycoside, MAC = macrolide. White space: not tested (only resistance phenotypes predicted *in silico* was tested *in vitro*).

Supplementary Table 2. Metatranscriptome datasets used in this study (data collected in May 2019).

Run	Spots (M)	Bases	Size	GC content (%)	Project	Experiment	Biosample	Organism
SRR3257011	29.5	5.9Gbp	4.3G	47.9	PRJNA275012	SRX1647089	SAMN04569317	beef cattle
SRR3256999	39.6	7.9Gbp	5.7G	44.8	PRJNA275012	SRX1646944	SAMN04569304	beef cattle
SRR8050516	2.5	485.7Mbp	322.0M	43.4	PRJNA496209	SRX4880196	SAMN10237315	beef cattle
SRR8050514	2.4	469.3Mbp	310.2M	42.4	PRJNA496209	SRX4880192	SAMN10237319	beef cattle
SRR8050517	2.1	399.9Mbp	273.7M	41.1	PRJNA496209	SRX4880189	SAMN10237324	beef cattle
SRR3169847	12.1	4.6Gbp	2.6G	51	PRJNA312137	SRX1585086	SAMN04494890	dairy cow
SRR3169851	10.5	3.9Gbp	2.2G	49.5	PRJNA312137	SRX1585089	SAMN04494891	dairy cow
SRR594654	14.3	2.9Gbp	1.8G	48.7	PRJNA176598	SRX196410	SAMN01766952	dairy cow
SRR5805524	62.9	15.7Gbp	5.6G	48.9	PRJNA393042	SRX2984721	SAMN07313972	dairy cow
SRR5805529	53.3	13.3Gbp	4.8G	46.6	PRJNA393042	SRX2984716	SAMN07313979	dairy cow
SRR6201277	8.2	2.5Gbp	1.6G	46.3	PRJNA366601	SRX3311193	SAMN06268723	sheep
SRR6201276	8.1	2.4Gbp	1.6G	46.3	PRJNA366601	SRX3311194	SAMN06268723	sheep
SRR873462	13.6	4.1Gbp	2.7G	46.7	PRJNA202380	SRX286794	SAMN02144231	sheep
SRR1138697	22.4	6.7Gbp	4.2G	50.7	PRJNA202380	SRX445849	SAMN02144230	sheep
SRR873454	10.4	3.1Gbp	2G	51	PRJNA202380	SRX286786	SAMN02144230	sheep
