# Supplementary material for: Characterization of antibiotic resistance genes in the species of the rumen microbiota
Source: Nat Commun. 2019 Nov 20;10:5252. doi: 10.1038/s41467-019-13118-0 (PMC6868206; doi:10.1038/s41467-019-13118-0)
Supplement: Supplementary file 2 — Description of Additional Supplementary Files [file 41467_2019_13118_MOESM2_ESM.docx]

**Description of Additional Supplementary Files**

File Name: Supplementary Data 1

Genetic context of the *tet*(W) gene distributed in ruminal genomes. The genes are represented by arrows classified and colored according to their function. Blue: phage proteins, yellow: methyltransferases; green: transposon/integron proteins; pink: conjugations proteins; red: *tet*(W); purple: other ARGs; brown: recombinase/endonuclease/excisionase; beige: Maff-2 protein, gray: genes encoding other proteins. The graphic representation of these flanking genes was performed using the software SnapGene 2.3.2.

File Name: Supplementary Data 2

Description: Alignment of the *Blautia schinkii* DSM 10518 genome and the DNA sequence of the novel ICE_*RbtetW*_*07* (covering the region from VirD4 gene to the *tet*(W) gene) obtained after PCR amplification and shotgun sequencing. Sequence alignment was performed using Clustal Omega.

File Name: Supplementary Data 3

Description: Genomes of ruminal bacteria and archaea used in this study (data collected from March 2017 to August 2017).
