# Supplementary figures and images for: Characterization of antibiotic resistance genes in the species of the rumen microbiota
Source: Nat Commun. 2019 Nov 20;10:5252. doi: 10.1038/s41467-019-13118-0 (PMC6868206; doi:10.1038/s41467-019-13118-0)

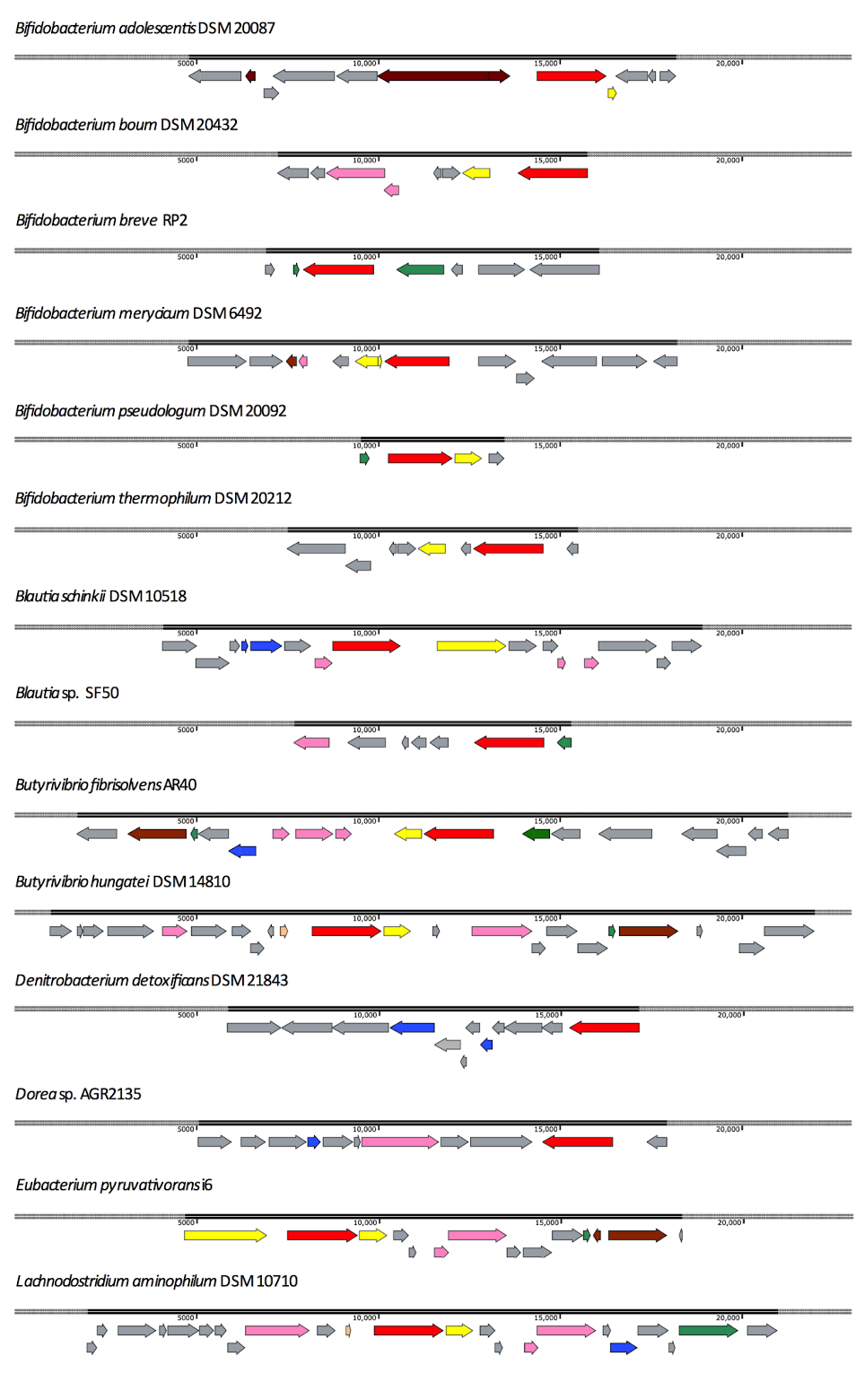


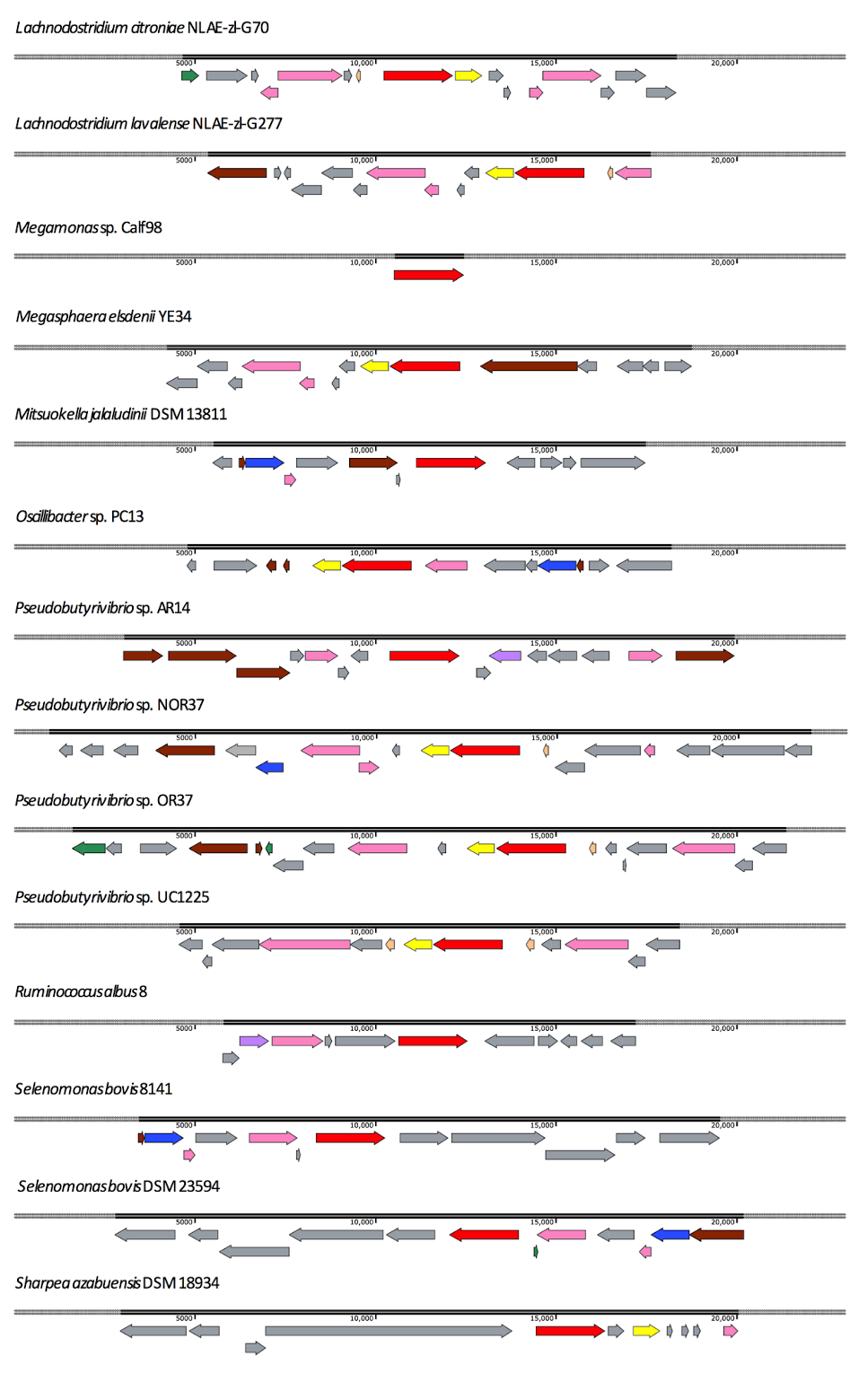

Supplement: Supplementary file 3 — Supplementary Data 1 [file 41467_2019_13118_MOESM3_ESM.docx]
