# Supplementary material for: Characterization of antibiotic resistance genes in the species of the rumen microbiota
Source: Nat Commun. 2019 Nov 20;10:5252. doi: 10.1038/s41467-019-13118-0 (PMC6868206; doi:10.1038/s41467-019-13118-0)
Supplement: Supplementary file 4 — Supplementary Data 2 [file 41467_2019_13118_MOESM4_ESM.docx]

Blautia_schinkii_DSM10518_Genome ATGAAAAAGCAGCTTGACATCAAAAAGCTCCTGATTTTGAACCTGCCCTATATCCTGATG 60

ICE_RbtetW_07

-----AAAGCAGCTTGACATCAAAAAGCTCCTGATTTTGAACCTGCCCTATATCCTGATG 55

*******************************************************

Blautia_schinkii_DSM10518_Genome GGCTTGTTTGCCACCAACTTCGGGGAAGCATGGCGGATGGCGCAGGGCGCGGACGCTTCT 120

ICE_RbtetW_07 GGCTTGTTTGCCACCAACTTCGGGGAAGCATGGCGGATGGCGCAGGGCGCGGACGCTTCT 115 ************************************************************

Blautia_schinkii_DSM10518_Genome CAAAAGGCGCTCTCCCTGATTTCTGTCTTGCCGGTGGCGCTGGCAAGCTGGTGGCCCAGC 180

ICE_RbtetW_07 CAAAAGGCGCTCTCCCTGATTTCTGTCTTGCCGGTGGCGCTGGCAAGCTGGTGGCCCAGC 175 ************************************************************

Blautia_schinkii_DSM10518_Genome CTGCACCCGTTGGACCTGTTGGTGGGAATCTGCTGCGGTGGTGGTCTGCGGCTGGCGGTA 240

ICE_RbtetW_07 CTGCACCCGTTGGACCTGTTGGTGGGAATCTGCTGCGGTGGTGGTCTGCGGCTGGCGGTA 235 ************************************************************

Blautia_schinkii_DSM10518_Genome TATCTGAAAAGCAAAAATGCGAAGAAATACCGCCACGGCATGGAGTATGGTTCCGCCCGC 300

ICE_RbtetW_07 TATCTGAAAAGCAAAAATGCGAAGAAATACCGCCACGGCATGGAGTATGGTTCCGCCCGC 295 ************************************************************

Blautia_schinkii_DSM10518_Genome TGGGGAACACACGAGGACATCGCCCCTTATGTGGACCCGGTTTTCCAGAACAATGTGATT 360

ICE_RbtetW_07 TGGGGAACACACGAGGACATCGCCCCTTATGTGGACCCGGTTTTCCAGAACAATGTGATT 355 ************************************************************

Blautia_schinkii_DSM10518_Genome CTGACGAAAACCGAGAGCCTGACCATGAACAGCCGCCCCAAGGACCCCAAGACGGCGCGA 420

ICE_RbtetW_07 CTGACGAAAACCGAGAGCCTGACCATGAACAGCCGCCCCAAGGACCCCAAGACGGCGCGA 415 ************************************************************

Blautia_schinkii_DSM10518_Genome AATAAAAATGTGCTGGTGATCGGCGGCTCCGGTTCCGGTAAAACACGGTTTTGGCTGAAA 480

ICE_RbtetW_07 AATAAAAATGTGCTGGTGATCGGCGGCTCCGGTTCCGGTAAAACACGGTTTTGGCTGAAA 475 ************************************************************

Blautia_schinkii_DSM10518_Genome CCCAACCTGATGCAGATGCACAGCTCCTATGTGGTGACAGACCCCAAAGGTACGATTTTG 540

ICE_RbtetW_07 CCCAACCTGATGCAGATGCACAGCTCCTATGTGGTGACAGACCCCAAAGGTACGATTTTG 535 ************************************************************

Blautia_schinkii_DSM10518_Genome GTGGAGTGCGGCAAGATGCTCCAGCGCGGTACGCCGAAAATGCGTCCCAAGCTGGGCAAG 600

ICE_RbtetW_07 GTGGAGTGCGGCAAGATGCTCCAGCGCGGTACGCCGAAAATGCGTCCCAAGCTGGGCAAG 595 ************************************************************

Blautia_schinkii_DSM10518_Genome GACCATCAGCCAATAAGGGACCGGCATGGCAATCCGGTTTATGAGACCGTAAAGGACAAA 660

ICE_RbtetW_07 GACCATCAGCCAATAAGGGACCGGCATGGCAATCCGGTTTATGAGACCGTAAAGGACAAA 655 ************************************************************

Blautia_schinkii_DSM10518_Genome AACGGCAAAGTGGTCTATGAGCCGTATCGAATTAAGGTTCTCAATACCATCAACTTCAAG 720

ICE_RbtetW_07 AACGGCAAAGTGGTCTATGAGCCGTATCGAATTAAGGTTCTCAATACCATCAACTTCAAG 715 ************************************************************

Blautia_schinkii_DSM10518_Genome AAGTCCATGCACTATAATCCTTTTGCCTATCTGCACAGCGAAAAAGATATTTTGAAGCTG 780

ICE_RbtetW_07 AAGTCCATGCACTATAATCCTTTTGCCTATCTGCACAGCGAAAAAGATATTTTGAAGCTG 775 ************************************************************

Blautia_schinkii_DSM10518_Genome GTCACGACTTTAATAGCGAACACCAAGGGAGAAGGCAAAGCCGGGGACGATTTCTGGGTC 840

ICE_RbtetW_07 GTCACGACTTTAATAGCGAACACCAAGGGAGAAGGCAAAGCCGGGGACGATTTCTGGGTC 835 ************************************************************

Blautia_schinkii_DSM10518_Genome AAGGCAGAAACGCTTTTGTACTGCGCCCTCATCGGATATATCCACTACGAGGCCCCGGTG 900

ICE_RbtetW_07 AAGGCAGAAACGCTTTTGTACTGCGCCCTCATCGGATATATCCACTACGAGGCCCCGGTG 895 ************************************************************

Blautia_schinkii_DSM10518_Genome GAAGAACAAAACTTTGCCACTCTCATCGAGTTCATCAACGCGATGGAAGTCCGGGAGGAC 960

ICE_RbtetW_07 GAAGAACAAAACTTTGCCACTCTCATCGAGTTCATCAACGCGATGGAAGTCCGGGAGGAC 955 ************************************************************

Blautia_schinkii_DSM10518_Genome GACGAGGAGTTCAAGAATCCGGTGGACCTGATGTTTGACGCACTGGAAGCGGAAAAGCCC 1020

ICE_RbtetW_07 GACGAGGAGTTCAAGAATCCGGTGGACCTGATGTTTGACGCACTGGAAGCGGAAAAGCCC 1015 ************************************************************

Blautia_schinkii_DSM10518_Genome AATCATTTTGCCGTCCGCCAATATAAAAAATACAAGCTGGCGGCTGGCAAAACCGCAAAA 1080

ICE_RbtetW_07 AATCATTTTGCCGTCCGCCAATATAAAAAATACAAGCTGGCGGCTGGCAAAACCGCAAAA 1075 ************************************************************

Blautia_schinkii_DSM10518_Genome TCCATTCTGATTTCCTGCGGTGCGCGCCTTGCCGTATTCGACATTGCGGAACTGCGGGAG 1140

ICE_RbtetW_07 TCCATTCTGATTTCCTGCGGTGCGCGCCTTGCCGTATTCGACATTGCGGAACTGCGGGAG 1135 ************************************************************

Blautia_schinkii_DSM10518_Genome GTCACTTCCTACGACGAGCTGGAGCTGGACACCCTGGGAGATCGGAAAACCGCCCTGTTC 1200

ICE_RbtetW_07 GTCACTTCCTACGACGAGCTGGAGCTGGACACCCTGGGAGATCGGAAAACCGCCCTGTTC 1195 ************************************************************

Blautia_schinkii_DSM10518_Genome CTCATTATGAGCGACACGGACGATAGCTTTAACTTCCTGATCTCCATGTGCTACACCCAG 1260

ICE_RbtetW_07 CTCATTATGAGCGACACGGACGATAGCTTTAACTTCCTGATCTCCATGTGCTACACCCAG 1255 ************************************************************

Blautia_schinkii_DSM10518_Genome CTGTTCAACCTTTTGTGTGAAAAAGCCGACGATGTGTACGGCGGGCGGTTGCCGGTCCAT 1320

ICE_RbtetW_07 CTGTTCAACCTTTTGTGTGAAAAAGCCGACGATGTGTACGGCGGGCGGTTGCCGGTCCAT 1315 ************************************************************

Blautia_schinkii_DSM10518_Genome GTGCGCTGCCTCATTGACGAGTGTGCCAACATCGGCCAGATTCCCAAGCTGGAAAAACTG 1380

ICE_RbtetW_07 GTGCGCTGCCTCATTGACGAGTGTGCCAACATCGGCCAGATTCCCAAGCTGGAAAAACTG 1375 ************************************************************

Blautia_schinkii_DSM10518_Genome GTCGCCACCATCCGCAGCCGTGAGATCTCCGCCTGTCTGGTATTGCAGGCGCAGTCCCAG 1440

ICE_RbtetW_07 GTCGCCACCATCCGCAGCCGTGAGATCTCCGCCTGTCTGGTATTGCAGGCGCAGTCCCAG 1435 ************************************************************

Blautia_schinkii_DSM10518_Genome CTGAAAGCCATCTACAAGGACAACGCCGATACCATCATCGGCAACATGGATACCTCCATC 1500

ICE_RbtetW_07 CTGAAAGCCATCTACAAGGACAACGCCGATACCATCATCGGCAACATGGATACCTCCATC 1495 ************************************************************

Blautia_schinkii_DSM10518_Genome TTCCTGGGCGGCAAGGAACCGACTACCCTCAAGGAGCTGGCCGCCGTGCTGGGCAAGGAA 1560

ICE_RbtetW_07 TTCCTGGGCGGCAAGGAACCGACTACCCTCAAGGAGCTGGCCGCCGTGCTGGGCAAGGAA 1555 ************************************************************

Blautia_schinkii_DSM10518_Genome ACCATCGACACCTACAACACCGGCGAGAACCGTGGGCGGGAAACCTCCCACTCTCTCAAC 1620

ICE_RbtetW_07 ACCATCGACACCTACAACACCGGCGAGAACCGTGGGCGGGAAACCTCCCACTCTCTCAAC 1615 ************************************************************

Blautia_schinkii_DSM10518_Genome TACCAGAAGCTCGGCAAAGAGCTTATGAGCCAGGATGAACTGGCCGTTATGGACGGCGGC 1680

ICE_RbtetW_07 TACCAGAAGCTCGGCAAAGAGCTTATGAGCCAGGATGAACTGGCCGTTATGGACGGCGGC 1675 ************************************************************

Blautia_schinkii_DSM10518_Genome AAGTGCATCCTCCAACTGCGCGGTGTGCGGCCTTTCCTCTCGGACAAGTACGACATCACC 1740

ICE_RbtetW_07 AAGTGCATCCTCCAACTGCGCGGTGTGCGGCCTTTCCTCTCGGACAAGTACGACATCACC 1735 ************************************************************

Blautia_schinkii_DSM10518_Genome AAGCACCCCAATTACCCGTACACCGCCGACGCGGACCCCAAGAACGCCTTTGACATCGAG 1800

ICE_RbtetW_07 AAGCACCCCAATTACCCGTACACCGCCGACGCGGACCCCAAGAACGCCTTTGACATCGAG 1795 ************************************************************

Blautia_schinkii_DSM10518_Genome GCGTTCCTGTCCACCCGGCTCAAGCTCAAGCCTAACGAGGTCTACGATGTGTATGAAGTA 1860

ICE_RbtetW_07 GCGTTCCTGTCCACCCGGCTCAAGCTCAAGCCTAACGAGGTCTACGATGTGTATGAAGTA 1855 ************************************************************

Blautia_schinkii_DSM10518_Genome GACGCAGAGGGCGCGTAAATCTGTTCCGCTGTGAAAGGAGTGATCTTATCTACCCGCCTC 1920

ICE_RbtetW_07 GACGCAGAGGGCGCGTAAATCTGTTCCGCTGTGAAAGGAGTGATCTTATCTACCCGCCTC 1915 ************************************************************

Blautia_schinkii_DSM10518_Genome AACCGCCCCGGATGGGGCCATCGGCGTACAAAGCGGACAATCACCAAAAAATAATGAAAA 1980

ICE_RbtetW_07 AACCGCCCCGGATGGGGCCATCGGCGTACAAAGCGGACAATCACCAAAAAATAATGAAAA 1975 ************************************************************

Blautia_schinkii_DSM10518_Genome AAGGAGGACAGCCGGAATCAGGCCCCGGAAACCGGGTGCCGATTGTTCCGGCTTTTGTAT 2040

ICE_RbtetW_07 AAGGAGGACAGCCGGAATCAGGCCCCGGAAACCGGGTGCCGATTGTTCCGGCTTTTGTAT 2035 ************************************************************

Blautia_schinkii_DSM10518_Genome GCCTATGAATGACTAAATCAAACTTTTCAAATGATTCCGGCTAACTATAATTTATGGCAT 2100

ICE_RbtetW_07 GCCTATGAATGACTAAATCAAACTTTTCAAATGATTCCGGCTAACTATAATTTATGGCAT 2095 ************************************************************

Blautia_schinkii_DSM10518_Genome TTTTCAATCAGGCTATCACCGTTCTTCAGACCCTCGTTATCGCTCTGGGCGCTGGTCTCG 2160

ICE_RbtetW_07 TTTTCAATCAGGCTATCACCGTTCTTCAGACCCTCGTTATCGCTCTGGGCGCTGGTCTCG 2155 ************************************************************

Blautia_schinkii_DSM10518_Genome GCATCTGGGGTGTCATCAACCTGCTGGAAGGTTACGGCAACGACAACCCCGGTGCGAATG 2220

ICE_RbtetW_07 GCATCTGGGGTGTCATCAACCTGCTGGAAGGTTACGGCAACGACAACCCCGGTGCGAATG 2215 ************************************************************

Blautia_schinkii_DSM10518_Genome CTCATGTACGGTAAGGAAGCAAGCAACCGAAAACAAGAGATAGACCGCCAGCACTACACT 2280

ICE_RbtetW_07 CTCATGTACGGTAAGGAAGCAAGCAACCGAAAACAAGAGATAGACCGCCAGCACTACACT 2275 ************************************************************

Blautia_schinkii_DSM10518_Genome ATTCCGAACCAAAGACCAAAAGATAAGCATTGTGGGAAAATCTAAACTTTTGGATTTTCC 2340

ICE_RbtetW_07 ATTCCGAACCAAAGACCAAAAGATAAGCATTGTGGGAAAATCTAAACTTTTGGATTTTCC 2335 ************************************************************

Blautia_schinkii_DSM10518_Genome TACAATGCCAACTACGGCGGAATCCCTCCCACTCCTTATATCTTTCTGTATACATTGAAT 2400

ICE_RbtetW_07 TACAATGCCAACTACGGCGGAATCCCTCCCACTCCTTATATCTTTCTGTATACATTGAAT 2395 ************************************************************

Blautia_schinkii_DSM10518_Genome TTGTATTTAGTAAAATGCAGACAACACCACGGATCGGCTTTTGGTTGGACAATTCCAACC 2460

ICE_RbtetW_07 TTGTATTTAGTAAAATGCAGACAACACCACGGATCGGCTTTTGGTTGGACAATTCCAACC 2455 ************************************************************

Blautia_schinkii_DSM10518_Genome AAACACCACAGCAGACAGCAGAAAACATTCTGAACGCTAGGAAGCCGGTATGATTGTTAC 2520

ICE_RbtetW_07 AAACACCACAGCAGACAGCAGAAAACATTCTGAACGCTAGGAAGCCGGTATGATTGTTAC 2515 ************************************************************

Blautia_schinkii_DSM10518_Genome ATATAAGGGGAAGAAAAATTTCTTTTAGGTACTTGCTTTCCTAAAACTGATGTGATACAA 2580

ICE_RbtetW_07 ATATAAGGGGAAGAAAAATTTCTTTTAGGTACTTGCTTTCCTAAAACTGATGTGATACAA 2575 ************************************************************

Blautia_schinkii_DSM10518_Genome TGATTTAATCCAGAAAAGGAGTAAAAAATATGCGGCAAGGTATTCTTAAATAAAACTATA 2640

ICE_RbtetW_07 TGATTTAATCCAGAAAAGGAGTAAAAAATATGCGGCAAGGTATTCTTAAATAAAACTATA 2635 ************************************************************

Blautia_schinkii_DSM10518_Genome ATCAAATAGTGGGAACAAAGGATTATGATAGCTCCTTTTGTAGGGGCTTAGTTTTTTGTA 2700

ICE_RbtetW_07 ATCAAATAGTGGGAACAAAGGATTATGATAGCTCCTTTTGTAGGGGCTTAGTTTTTTGTA 2695 ************************************************************

Blautia_schinkii_DSM10518_Genome CCCAATTTAAGAATACTTTTGCCTTATCAATTTTGACATATCCCCAAAAACAGCAATCAC 2760

ICE_RbtetW_07 CCCAATTTAAGAATACTTTTGCCTTATCAATTTTGACATATCCCCAAAAACAGCAATCAC 2755 ************************************************************

Blautia_schinkii_DSM10518_Genome AAACAGGTGTATGCTGTATATGTGTATGTCCGCAACTTATAATCCCCAGTGGTAAAAGTA 2820

ICE_RbtetW_07 AAACAGGTGTATGCTGTATATGTGTATGTCCGCAACTTATAATCCCCAGTGGTAAAAGTA 2815 ************************************************************

Blautia_schinkii_DSM10518_Genome TTTTACTGCTGGGGATTTTTATGCCCTTTGGGGCTGTAAAGGGAGGACAATCACATGAAA 2880

ICE_RbtetW_07 TTTTACTGCTGGGGATTTTTATGCCCTTTGGGGCTGTAAAGGGAGGACAATCACATGAAA 2875 ************************************************************

Blautia_schinkii_DSM10518_Genome ATAATCAATATTGGAATTCTTGCCCATGTAGACGCTGGAAAGACGACCTTGACGGAGAGC 2940

ICE_RbtetW_07 ATAATCAATATTGGAATTCTTGCCCATGTAGACGCTGGAAAGACGACCTTGACGGAGAGC 2935 ************************************************************

Blautia_schinkii_DSM10518_Genome CTGCTATATGCCAGCGGAGCCATTTCAGAACCGGGGAGCGTCGAAAAAGGGACAACGAGG 3000

ICE_RbtetW_07 CTGCTATATGCCAGCGGAGCCATTTCAGAACCGGGGAGCGTCGAAAAAGGGACAACGAGG 2995 ************************************************************

Blautia_schinkii_DSM10518_Genome ACGGACACCATGTTTTTGGAGCGGCAGCGTGGGATTACCATTCAAGCGGCAGTCACTTCC 3060

ICE_RbtetW_07 ACGGACACCATGTTTTTGGAGCGGCAGCGTGGGATTACCATTCAAGCGGCAGTCACTTCC 3055 ************************************************************

Blautia_schinkii_DSM10518_Genome TTCCAGTGGCACAGATGTAAAGTTAACATTGTGGATACGCCCGGCCACATGGATTTTTTG 3120

ICE_RbtetW_07 TTCCAGTGGCACAGATGTAAAGTTAACATTGTGGATACGCCCGGCCACATGGATTTTTTG 3115 ************************************************************

Blautia_schinkii_DSM10518_Genome GCGGAGGTGTACCGCTCTTTGGCTGTTTTAGATGGGGCCATCTTGGTGATCTCCGCTAAA 3180

ICE_RbtetW_07 GCGGAGGTGTACCGCTCTTTGGCTGTTTTAGATGGGGCCATCTTGGTGATCTCCGCTAAA 3175 ************************************************************

Blautia_schinkii_DSM10518_Genome GATGGCGTGCAGGCCCAGACCCGTATTCTGTTCCATGCCCTGCGGAAAATGAACATTCCC 3240

ICE_RbtetW_07 GATGGCGTGCAGGCCCAGACCCGTATTCTGTTCCATGCCCTGCGGAAAATGAACATTCCC 3235 ************************************************************

Blautia_schinkii_DSM10518_Genome ACCGTTATCTTTATCAACAAGATCGACCAGGCTGGCGTTGATTTGCAGAGCGTGGTTCAG 3300

ICE_RbtetW_07 ACCGTTATCTTTATCAACAAGATCGACCAGGCTGGCGTTGATTTGCAGAGCGTGGTTCAG 3295 ************************************************************

Blautia_schinkii_DSM10518_Genome TCTGTTCGGGATAAGCTCTCCGCCGATATTATCATCAAGCAGACGGTGTCGCTGTCCCCG 3360

ICE_RbtetW_07 TCTGTTCGGGATAAGCTCTCCGCCGATATTATCATCAAGCAGACGGTGTCGCTGTCCCCG 3355 ************************************************************

Blautia_schinkii_DSM10518_Genome GAAATAGTCCTGGAGGAAAATACCGACATAGAAGCATGGGATGCGGTCATCGAAAATAAC 3420

ICE_RbtetW_07 GAAATAGTCCTGGAGGAAAATACCGACATAGAAGCATGGGATGCGGTCATCGAAAATAAC 3415 ************************************************************

Blautia_schinkii_DSM10518_Genome GATGAATTATTGGAAAAGTATATCGCAGGAGAACCAATCAGCCGGGAAAAACTTGCGCGG 3480

ICE_RbtetW_07 GATGAATTATTGGAAAAGTATATCGCAGGAGAACCAATCAGCCGGGAAAAACTTGCGCGG 3475 ************************************************************

Blautia_schinkii_DSM10518_Genome GAGGAACAGCAGCGGGTTCAAGACGCCTCCCTGTTCCCAGTCTATCATGGCAGCGCCAAA 3540

ICE_RbtetW_07 GAGGAACAGCAGCGGGTTCAAGACGCCTCCCTGTTCCCAGTCTATCATGGCAGCGCCAAA 3535 ************************************************************

Blautia_schinkii_DSM10518_Genome AATGGCCTTGGCATTCAACCGTTGATGGATGCGGTGACAGGGCTGTTCCAACCGATTGGG 3600

ICE_RbtetW_07 AATGGCCTTGGCATTCAACCGTTGATGGATGCGGTGACAGGGCTGTTCCAACCGATTGGG 3595 ************************************************************

Blautia_schinkii_DSM10518_Genome GAACAGGGGGGCGCCGCCCTATGCGGCAGCGTTTTCAAGGTTGAGTACACCGATTGCGGC 3660

ICE_RbtetW_07 GAACAGGGGGGCGCCGCCCTATGCGGCAGCGTTTTCAAGGTTGAGTACACCGATTGCGGC 3655 ************************************************************

Blautia_schinkii_DSM10518_Genome CAGCGGCGTGTCTATCTACGGTTATACAGCGGAACGCTGCGCCTGCGGGATACGGTGGCC 3720

ICE_RbtetW_07 CAGCGGCGTGTCTATCTACGGTTATACAGCGGAACGCTGCGCCTGCGGGATACGGTGGCC 3715 ************************************************************

Blautia_schinkii_DSM10518_Genome CTGGCCGGGAGAGAAAAGCTGAAAATCACAGAGATGCGTATTCCATCCAAAGGGGAAATT 3780

ICE_RbtetW_07 CTGGCCGGGAGAGAAAAGCTGAAAATCACAGAGATGCGTATTCCATCCAAAGGGGAAATT 3775 ************************************************************

Blautia_schinkii_DSM10518_Genome GTTCGGACAGACACCGCTTATCAGGGTGAAATTGTTATCCTTCCCAGCGACAGCGTGAGG 3840

ICE_RbtetW_07 GTTCGGACAGACACCGCTTATCAGGGTGAAATTGTTATCCTTCCCAGCGACAGCGTGAGG 3835 ************************************************************

Blautia_schinkii_DSM10518_Genome TTAAACGATGTATTAGGGGACCAAACCCGGCTCCCTCGTAAAAGGTGGCGCGAGGACCCC 3900

ICE_RbtetW_07 TTAAACGATGTATTAGGGGACCAAACCCGGCTCCCTCGTAAAAGGTGGCGCGAGGACCCC 3895 ************************************************************

Blautia_schinkii_DSM10518_Genome CTCCCCATGCTGCGGACGACGATTGCGCCGAAAACGGCAGCGCAAAGAGAACGGCTGCTG 3960

ICE_RbtetW_07 CTCCCCATGCTGCGGACGACGATTGCGCCGAAAACGGCAGCGCAAAGAGAACGGCTGCTG 3955 ************************************************************

Blautia_schinkii_DSM10518_Genome GACGCTCTTACGCAACTTGCGGATACTGACCCGCTTTTGCGTTGCGAAGTGGATTCCATC 4020

ICE_RbtetW_07 GACGCTCTTACGCAACTTGCGGATACTGACCCGCTTTTGCGTTGCGAAGTGGATTCCATC 4015 ************************************************************

Blautia_schinkii_DSM10518_Genome ACCCATGAGATCATTCTTTCTTTTTTGGGCCGGGTGCAGTTGGAGGTTGTTTCCGCTTTG 4080

ICE_RbtetW_07 ACCCATGAGATCATTCTTTCTTTTTTGGGCCGGGTGCAGTTGGAGGTTGTTTCCGCTTTG 4075 ************************************************************

Blautia_schinkii_DSM10518_Genome CTGTCGGAAAAATACAAGCTTGAAACAGTGGTAAAGGAACCCTCCGTCATTTATATGGAG 4140

ICE_RbtetW_07 CTGTCGGAAAAATACAAGCTTGAAACAGTGGTAAAGGAACCCTCCGTCATTTATATGGAG 4135 ************************************************************

Blautia_schinkii_DSM10518_Genome CGGCCGCTCAAAGCAGCCAGCCACACCATCCATATCGAGGTGCCGCCCAACCCGTTTTGG 4200

ICE_RbtetW_07 CGGCCGCTCAAAGCAGCCAGCCACACCATCCATATCGAGGTGCCGCCCAACCCGTTTTGG 4195 ************************************************************

Blautia_schinkii_DSM10518_Genome GCATCCATAGGACTGTCTGTTACACCACTCTCGCTTGGCTCCGGTGTACAATACGAGAGC 4260

ICE_RbtetW_07 GCATCCATAGGACTGTCTGTTACACCACTCTCGCTTGGCTCCGGTGTACAATACGAGAGC 4255 ************************************************************

Blautia_schinkii_DSM10518_Genome CGGGTTTCGCTGGGATACTTGAACCAGAGTTTTCAAAACGCTGTCAGGGATGGTATCCGT 4320

ICE_RbtetW_07 CGGGTTTCGCTGGGATACTTGAACCAGAGTTTTCAAAACGCTGTCAGGGATGGTATCCGT 4315 ************************************************************

Blautia_schinkii_DSM10518_Genome TACGGGCTGGAGCAGGGCTTGTTCGGCTGGAACGTAACGGACTGTAAGATTTGCTTTGAA 4380

ICE_RbtetW_07 TACGGGCTGGAGCAGGGCTTGTTCGGCTGGAACGTAACGGACTGTAAGATTTGCTTTGAA 4375 ************************************************************

Blautia_schinkii_DSM10518_Genome TACGGGCTTTATTACAGTCCGGTCAGCACGCCGGCGGACTTCCGCTCATTGGCCCCGATT 4440

ICE_RbtetW_07 TACGGGCTTTATTACAGTCCGGTCAGCACGCCGGCGGACTTCCGCTCATTGGCCCCGATT 4435 ************************************************************

Blautia_schinkii_DSM10518_Genome GTATTGGAACAGGCATTGAAGGAATCGGGGACGCAGCTGCTGGAACCTTATCTCTCCTTC 4500

ICE_RbtetW_07 GTATTGGAACAGGCATTGAAGGAATCGGGGACGCAGCTGCTGGAACCTTATCTCTCCTTC 4495 ************************************************************

Blautia_schinkii_DSM10518_Genome ATCCTCTATGCGCCCCAGGAATACCTTTCCAGGGCTTATCATGATGCACCGAAATACTGT 4560

ICE_RbtetW_07 ATCCTCTATGCGCCCCAGGAATACCTTTCCAGGGCTTATCATGATGCACCGAAATACTGT 4555 ************************************************************

Blautia_schinkii_DSM10518_Genome GCCACCATCGAAACGGCCCAGGTAAAAAAGGATGAAGTTGTCTTTACTGGCGAGATTCCC 4620

ICE_RbtetW_07 GCCACCATCGAAACGGCCCAGGTAAAAAAGGATGAAGTTGTCTTTACTGGCGAGATTCCC 4615 ************************************************************

Blautia_schinkii_DSM10518_Genome GCCCGCTGTATACAGGCATACCGTACTGATCTGGCCTTTTACACCAACGGGCGGAGCGTA 4680

ICE_RbtetW_07 GCCCGCTGTATACAGGCATACCGTACTGATCTGGCCTTTTACACCAACGGGCGGAGCGTA 4675 ************************************************************

Blautia_schinkii_DSM10518_Genome TGCCTTACAGAGCTGAAAGGATATCAGGCCGCTGTCGGTCAGCCGGTCATCCAGCCCCGC 4740

ICE_RbtetW_07 TGCCTTACAGAGCTGAAAGGATATCAGGCCGCTGTCGGTCAGCCGGTCATCCAGCCCCGC 4735 ************************************************************

Blautia_schinkii_DSM10518_Genome CGTCCAAACAGCCGCCTGGACAAGGTGCGCCATATGTTTCAGAAGGTAATGTAA 4794

ICE_RbtetW_07 CGTCCAAACAGCCGCCTGGACAAGGTGCGCCATATGTTTCAGAAGGT------- 4782 ***********************************************
