# Supplementary material for: Characterization of antibiotic resistance genes in the species of the rumen microbiota
Source: Nat Commun. 2019 Nov 20;10:5252. doi: 10.1038/s41467-019-13118-0 (PMC6868206; doi:10.1038/s41467-019-13118-0)
Supplement: Supplementary file 6 — Reporting Summary [file 41467_2019_13118_MOESM6_ESM.pdf]

nature research

Corresponding author(s): Huws, S.A.; Mantovani, H.C.

Last updated by author(s): Oct 9, 2019

Reporting Summary

Nature Research wishes to improve the reproducibility of the work that we publish. This form provides structure for consistency and transparency in reporting. For further information on Nature Research policies, see Authors & Referees and the Editorial Policy Checklist.

Statistics

For all statistical analyses, confirm that the following items are present in the figure legend, table legend, main text, or Methods section.

n/a	Confirmed	
☐	☒	The exact sample size (*n*) for each experimental group/condition, given as a discrete number and unit of measurement
☐	☒	A statement on whether measurements were taken from distinct samples or whether the same sample was measured repeatedly
☐	☒	The statistical test(s) used AND whether they are one- or two-sided<br>*Only common tests should be described solely by name; describe more complex techniques in the Methods section.*
☐	☒	A description of all covariates tested
☒	☐	A description of any assumptions or corrections, such as tests of normality and adjustment for multiple comparisons
☒	☐	A full description of the statistical parameters including central tendency (e.g. means) or other basic estimates (e.g. regression coefficient) AND variation (e.g. standard deviation) or associated estimates of uncertainty (e.g. confidence intervals)
☐	☒	For null hypothesis testing, the test statistic (e.g. *F*, *t*, *r*) with confidence intervals, effect sizes, degrees of freedom and *P* value noted<br>*Give P values as exact values whenever suitable.*
☒	☐	For Bayesian analysis, information on the choice of priors and Markov chain Monte Carlo settings
☒	☐	For hierarchical and complex designs, identification of the appropriate level for tests and full reporting of outcomes
☒	☐	Estimates of effect sizes (e.g. Cohen's *d*, Pearson's *r*), indicating how they were calculated

Our web collection on statistics for biologists contains articles on many of the points above.

Software and code

Policy information about availability of computer code

Data collection: Data for this study was obtained directly from the NCBI and JGI websites and from the Hungate1000 Project. No software was used to collect the data.

Data analysis: To analyze the data, the following softwares were used: Resfinder v2.1, Resfams v1.2, Prodigal v1.2, ARG-ANNOT Nt V3, RDP Release 11.5 aligner, FastTree v2.1, Interactive Tree of Life (iTOL) v4, Prokka v1.12, Galaxy plataform, Samtools v1.9, Muscle 3.8.31, Uniprot, ISFinder, PlasmidFinder 2.0, PHASTER, ICEBerg 2.0, MAUVE, MegaX, JCoDA v1.4, DnaSP v6, FastQC v0.11.5, Bowtie2, Trimmomatic v0.27, SPAdes v3.10.1.

For manuscripts utilizing custom algorithms or software that are central to the research but not yet described in published literature, software must be made available to editors/reviewers. We strongly encourage code deposition in a community repository (e.g. GitHub). See the Nature Research guidelines for submitting code & software for further information.

Data

Policy information about availability of data

All manuscripts must include a data availability statement. This statement should provide the following information, where applicable:
- Accession codes, unique identifiers, or web links for publicly available datasets
- A list of figures that have associated raw data
- A description of any restrictions on data availability

The data that support the findings of this study are included in the paper and/or its supplementary information files. Data underlying Figures 5, 6, 8 and Supplementary Figures 1, 2 and 9 are provided as a Source Data file.

Field-specific reporting

Please select the one below that is the best fit for your research. If you are not sure, read the appropriate sections before making your selection.

☒ Life sciences ☐ Behavioural & social sciences ☐ Ecological, evolutionary & environmental sciences

For a reference copy of the document with all sections, see nature.com/documents/nr-reporting-summary-flat.pdf

Life sciences study design

All studies must disclose on these points even when the disclosure is negative.

Sample size	All available genomes belonging to the Hungate1000 collection (435 genomes) were selected for mining of antibiotic research genes (ARGs). Bacterial cultures available in the DSMZ culture catalogue harboring ARGs detected in our in silico analyses (total of 26 cultures) were selected for the in vitro analyses.
Data exclusions	Only complete genomes that were publicly available were used in our in silico analyses. Incomplete (draft) genomes were excluded.
Replication	The in vitro analysis were performed in duplicate measurements and with three biological replicates. Reported results represent the average of these values.
Randomization	The in vitro experiments followed a completely randomized design for the evaluation of minimum inhibitory concentrations (MIC).
Blinding	Blinding was not relevant in this study because the objective was to perform genome mining (all genomes available for the purpose of these analyses were used in this study). Therefore, group allocation was not used for data collection.

Reporting for specific materials, systems and methods

We require information from authors about some types of materials, experimental systems and methods used in many studies. Here, indicate whether each material, system or method listed is relevant to your study. If you are not sure if a list item applies to your research, read the appropriate section before selecting a response.

Materials & experimental systems

n/a	Involved in the study
☒	☐ Antibodies
☒	☐ Eukaryotic cell lines
☒	☐ Palaeontology
☒	☐ Animals and other organisms
☒	☐ Human research participants
☒	☐ Clinical data

Methods

n/a	Involved in the study
☒	☐ ChIP-seq
☒	☐ Flow cytometry
☒	☐ MRI-based neuroimaging
